# Combination of Early Allograft Dysfunction and Protein Expression Patterns Predicts Outcome of Liver Transplantation From Donation After Cardiac Death

**DOI:** 10.3389/fmed.2021.775212

**Published:** 2021-12-08

**Authors:** Qiang Wei, Junbin Zhou, Kun Wang, Xuanyu Zhang, Junli Chen, Di Lu, Xuyong Wei, Shusen Zheng, Xiao Xu

**Affiliations:** ^1^Department of Hepatobiliary and Pancreatic Surgery, The Center for Integrated Oncology and Precision Medicine, Affiliated Hangzhou First People's Hospital, Zhejiang University School of Medicine, Hangzhou, China; ^2^Institute of Organ Transplantation, Zhejiang University, Hangzhou, China; ^3^National Health Commission Key Laboratory of Combined Multi-Organ Transplantation, Hangzhou, China; ^4^Nanjing Drum Tower Hospital, The Affiliated Hospital of Nanjing University Medical School, Nanjing, China; ^5^Department of Hepatobiliary and Pancreatic Surgery, First Affiliated Hospital, Zhejiang University School of Medicine, Hangzhou, China; ^6^China Liver Transplant Registery, Hangzhou, China

**Keywords:** hepatic, liver transplant, EAD, protein profiles, prognosis

## Abstract

Early allograft dysfunction (EAD) after liver transplantation (LT) accompanies poor prognosis. This study aims to explore the relationship between pretransplant intrahepatic proteins and the incidence of EAD, and the value of combined EAD and protein profiles for predicting recipient and graft survival prognosis. Liver biopsy specimens of 105 pretransplant grafts used for LT were collected and used for immunohistochemistry analysis of 5 proteins. And matched clinical data of donor, recipient, transplantation, and prognosis were analyzed. The incidence of EAD was 41.9% (44/105) in this cohort. Macrovesicular steatosis (*P* = 0.016), donor body mass index (*P* = 0.013), recipients' pretransplant serum creatinine (*P* = 0.036), and intrahepatic expression of heme oxygenase 1 (HO1) (*P* = 0.015) and tumor necrosis factor α (TNF-α) (*P* = 0.039) were independent predictors of EAD. Inferior graft and recipient prognosis were observed in patients who experienced EAD (*P* = 0.028 and 0.031) or received grafts with higher expression of sirtuin 1 (*P* = 0.005 and 0.013). The graft and recipient survival were worst in patients with both EAD and high expression of sirtuin 1 (*P* = 0.001 and 0.004). In conclusion, pretransplant intrahepatic expression of HO1 and TNF-α are associated with the incidence of EAD. The combination of EAD and EAD-unrelated proteins showed superiority in distinguishing recipients with worse prognosis.

## Introduction

Liver transplantation (LT) has been accepted as the only available curative treatment for patients with end-stage liver disease. With rapidly increasing demand for LT, there is a serious shortage of donor organs. This has promoted the usage of marginal or extended criteria donors such as donation after cardiac death (DCD), steatosis, and senile donor livers. However, the use of these suboptimal grafts leads to higher morbidity and mortality of recipients.

Early allograft dysfunction (EAD) is a common complication following LT, with an incidence range between 20 and 40% (1). In recent years, the usage of marginal or extended criteria donors have increased the incidence of EAD (2). Although EAD is reversible, it could also deteriorate into primary non-function, demanding re-transplantation or leading to death. Recipients or grafts experiencing EAD tend to have an inferior survival prognosis (3, 4).

Up to now, there is still no widely acceptable approach for preventing or treating EAD. Many factors could contribute to the onset of EAD, such as donor age, donor body mass index (BMI), graft steatosis, cold ischemic time (CIT), warm ischemic time (WIT), donor serum sodium, and model for end-stage liver disease (MELD) score (5). Recently, multiple molecular markers of EAD have been introduced and improved the predicting profiles of EAD, such as microRNAs, circRNAs, and serum proteins (6–9). However, the role of protein expression patterns in pretransplant liver grafts in predicting the onset of EAD and recipients' prognosis remains poorly studied. Previous experimental studies have showed important roles of several intrahepatic proteins in liver injury during LT. For instance, hypoxia inducible factors (HIF) respond to the anoxic process during ischemia-reperfusion injury (IRI), and play a vital role in regulating inflammation, cell metabolism, and fibrosis (10); heme oxygenase (HO) 1 and sirtuin (SIRT) 1 were reported to be protective factors of liver function (11); toll-like receptor (TLR) 4 tends to harm liver function through promoting inflammation and inducing gut-liver axis-related liver injury (10); tumor necrosis factor (TNF) α might activate NF-κB-associated inflammation and IRI (12). However, the clinical application of these proteins remains to be explored.

In the present study, we aim to explore the relationship between the expression level of the above pretransplant intrahepatic proteins and the onset of EAD, and to explore the predictive value for graft and recipient survival prognosis of the combination of EAD and protein profiles.

## Patients and Methods

### Study Population and Data Collection

A total of 561 patients who underwent DCD LT in the First Affiliated Hospital, Zhejiang University School of Medicine from January 2015 to July 2017 were enrolled. We collected clinical information including donor and recipient demographics, blood type, BMI, steatotic status of donor liver, donor hepatitis B virus (HBV) infection, pretransplant recipient MELD score and serum creatinine, operation time, CIT, WIT, blood loss, postoperative intensive care unit (ICU) length, and ventilator support time. The patients' condition should be stably maintained without the need for major interventions such as transcatheter arterial chemoembolization or an artificial liver support system during the interval between pretransplant tests and the day of LT, otherwise the case would be excluded. After the exclusion of pediatric LT, multiple-organ transplantation, split LT, re-transplantation, and missing pretransplant biopsy specimens or essential data, 105 cases were finally enrolled into our study. The median of follow-up duration was 16.3 months.

EAD was defined as one or more of the following criteria: (1) serum bilirubin ≥ 10 mg/dL; (2) international normalized ratio (INR) ≥ 1.6 on posttransplant day 7; and (3) serum levels of aspartate aminotransferase (AST) or alanine aminotransferase (ALT) > 2,000 U/L within 7 days after LT. Each recipient was classified as “EAD” or “non-EAD” according to these criteria.

### Procurement of Grafts From Donors

The procurement of donor grafts was performed in accordance with the national guidelines for DCD in China. The procedure has been described in our previous studies (4). Briefly, DCD grafts were procured from donors and flushed with preservation solution. During the preparation period between cold storage and implantation of the grafts, a wedge specimen in the left lateral segment was routinely obtained for evaluation. Each organ donation or transplant was approved by the Institutional Review Board, Zhejiang University school of medicine (approval number: 2018-107), strictly under the guidelines of the Ethics Committee of the hospital, the current regulation of the Chinese Government, and the Declaration of Helsinki. Informed consent in writing was obtained from each patient. And no organs from executed prisoners were used.

### Immunohistochemical Staining and Evaluation

Immunohistochemistry (IHC) staining was performed according to a standard protocol (4). Five intrahepatic proteins were analyzed using IHC staining: HIF2-α, HO1, SIRT1, TLR4, and TNF-α. Staining intensity and area were evaluated for analyzing the expression level of proteins. And the product of intensity score and area score was calculated for defining the expression level of those five proteins (high or low expression). The cut-off value was determined according to the overall expression level of the corresponding proteins (13). Staining intensity was scored 0 (non), 1 (weak), 2 (moderate), and 3 (strong). Examples of the grading are shown in Supplementary Figure 1. Staining area was scored 0 (positivity < 5%), 1 (positivity between 5 and 25%), 2 (positivity between 25 and 50%), 3 (positivity between 50 and 75%), and 4 (positivity between 75 and 100%). At least three randomly chosen and non-overlapping fields (×200 magnification) were used for evaluation. The results were reviewed by two experienced pathologists who were blind to the study design.

### Statistical Analysis

The statistical analysis was performed using the statistical software IBM SPSS (Ver. 20.0; SPSS Inc., Chicago, IL, USA). Quantitative variables are expressed as the mean ± standard deviation or median and range depending on the distribution. Categorical variables are presented as values and percentages. Continuous variables were compared using the Mann-Whitney *U*-test or student's *t*-test. The chi-square test was used to compare categorical variables. Multivariate logistic regression analysis was performed to identify independent predictors of the onset of EAD, and the odds ratio (OR) was calculated. Survival analysis was performed to study the effects on graft and patient survival of each factors. All tests were two-sided, with a *P*-value of <0.05 considered statistically significant.

## Results

### Clinical Characteristics of Patients With or Without EAD

A total of 105 recipients who received primary LT were enrolled in this study. The incidence rate of EAD was 41.9% (44/105). The baseline characteristics of EAD and non-EAD recipients are summarized in Table 1.

**Table 1:** Summary of clinical characteristics of the study population.

	**Total (*n* = 105)**	**Non-EAD (*n* = 61)**	**EAD (*n* = 44)**	***P***
**Donor characteristics**				
Gender				0.871
Male	89 (84.8%)	52 (85.2%)	37 (84.1%)	
Female	16 (15.2%)	9 (14.8%)	7 (15.9%)	
Age, y^a^	40.0 ± 12.7	37.9 ± 12.9	42.9 ± 12.1	0.435
Height, cm^b^	168.0 (160.0–170.0)	167.5 (160.0–170.0)	168.0 (159.0–170.0)	0.621
Weight, kg^b^	65.0 (60.0–75.0)	65.0 (55.0-70.0)	67.0 (65.0–80.0)	0.056
Body mass index, kg/m^2b^	23.7 (21.5–26.1)	23.3 (20.8-25.0)	23.9 (22.5–27.3)	0.048
Macrovesicular steatosis				0.005
≥30%	20 (19.0%)	6 (9.8%)	14 (31.8%)	
<30%	85 (81.0%)	55 (90.2%)	30 (68.2)	
HBV infection				0.740
Positive	13 (12.4%)	7 (11.5%)	6 (13.6%)	
Negative	92 (87.6%)	54 (88.5%)	38 (86.4%)	
**Recipient characteristics**				
Gender				0.915
Male	83 (79.0%)	48 (78.7%)	35 (79.5%)	
Female	22 (21.0%)	13 (21.3%)	9 (20.5%)	
Age, y^a^	51.3 ± 9.6	52.2 ± 9.1	49.9 ± 10.3	0.450
Blood type (ABO)				0.208
Compatible	76 (72.4%)	47 (77.0%)	29 (65.9%)	
Incompatible	29 (27.6%)	14 (23.0%)	15 (34.1%)	
Height, cm^b^	170.0 (165.0–173.0)	170.0 (164.5–170.5)	170.0 (166.0–174.8)	0.101
Weight, kg^b^	65.0 (58.0–70.5)	64.0 (56.3–70.5)	65.0 (61.4–71.5)	0.168
Body mass index, kg/m^2b^	22.6 (20.9–24.2)	22.6 (20.2–24.2)	22.8 (21.5–24.1)	0.491
MELD^b^	18.0 (10.0–33.5)	15.0 (9.0–27.5)	24.0 (11.0–40.0)	0.006
Serum creatinine, μmol/L^b^	70.0 (59.0–108.0)	66.0 (58.5–81.0)	90.5 (60.5–186.3)	0.009
**Transplant characteristics**				
Cold ischemia time, h^b^	7.0 (5.7–10.5)	6.8 (5.3–8.5)	9.1 (6.0–12.6)	0.001
Warm ischemia time, min^b^	9.0 (3.0–14.0)	9.0 (3.0–13.5)	9.5 (5–14.8)	0.265
Operative time, h^b^	5.1 (4.5–5.7)	5.1 (4.5–5.7)	5.0 (4.5–5.8)	0.974
Blood loss, ml/kg^b^	13.3 (9.3–20.8)	12.2 (9.0–16.6)	15.2 (9.7–25.3)	0.051
**Post-transplantation**				
Intensive care unit length of stay, h^b^	200 (159.1–288.3)	207.0 (159.3–297.2)	197.8 (159.0–272.8)	0.678
Ventilator support time, h^b^	12.5 (8.5–17.5)	12.5 (8.0–16.1)	12.4 (9.3–30.3)	0.359

a *Mean ± SD*.

b *Median (25th−75th percentile)*.

*EAD, early allograft dysfunction*.

Through univariate analysis, the EAD group tended to have higher pretransplant levels of serum creatinine than the non-EAD group (90.5 vs. 66.0 μmol/L, *P* = 0.009), indicating that pretransplant renal function might affect the onset of EAD. Higher donor BMI rather than recipient BMI was associated with the development of EAD (*P* = 0.048 and 0.491, respectively). In addition, macrovesicular steatosis ≥ 30% (*P* = 0.005), higher MELD score (*P* = 0.006), and longer CIT (*P* = 0.001) were identified as risk factors associated with the onset of EAD. Donor/recipient age (*P* = 0.435 and 0.450, respectively), blood type compatibility (*P* = 0.208), and HBV infection (*P* = 0.740) were not associated with the onset of EAD in this analysis. The blood loss volume during operation in the EAD group was larger than that in the non-EAD group, although the difference was not statistically significant (15.2 vs. 12.2 ml/kg, *P* = 0.051).

### Protein Profiles of Donor Livers Correlated With EAD

Five proteins that might be correlated with the graft or recipient prognosis were identified by researching literature: HIF2-α, HO1, SIRT1, TLR4, and TNF-α. These proteins were then analyzed by IHC staining using pretransplant biopsy specimens. Examples of IHC staining of these five proteins are shown in Supplementary Figure 1. Cytoplasmic expression of HIF2-α, SIRT1, TLR4, and TNF-α was observed mostly in hepatocytes, while SIRT1 and TNF-α were mainly distributed in hepatocytes surrounding the central veins. Differently, IHC results showed that HO1 was mainly expressed in cells localized to the sinusoids.

**Figure 1:**
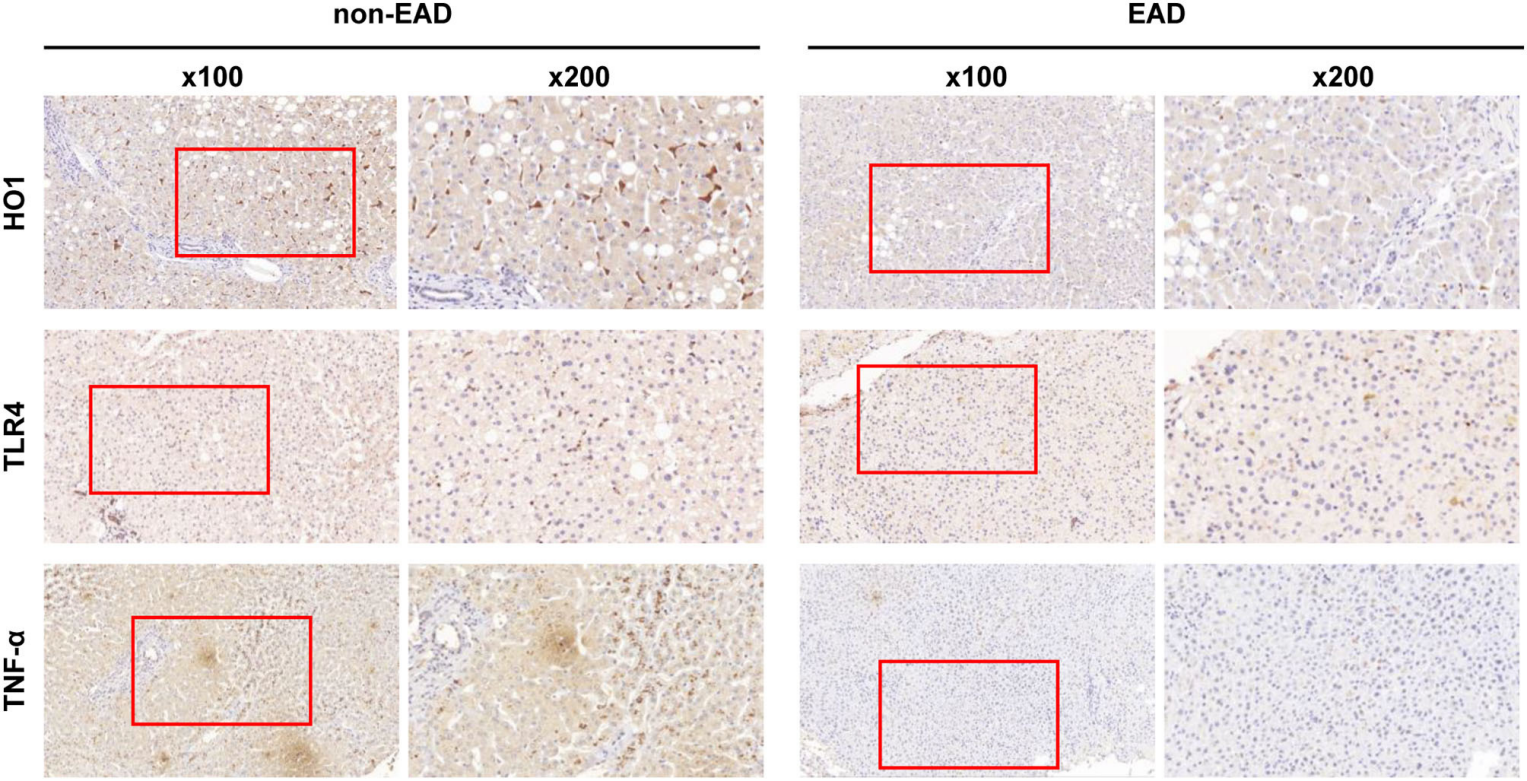
Representative immunohistochemical results of the intrahepatic expression of HO1, TLR4, and TNF-α between EAD (*n* = 44) and non-EAD (*n* = 61) groups. EAD, early allograft dysfunction; HO1, heme oxygenase-1; TLR4, toll like receptor 4; TNF-α, tumor necrosis factor α.

The expression levels of these proteins in liver tissues were compared between EAD and non-EAD groups by univariate analysis. Results showed that the EAD group had lower expression levels of TLR4 and TNF-α (*P* = 0.012 and 0.046, respectively) than the non-EAD group (Figure 1). However, the expression level of HIF2-α, HO1, or SIRT1 was not different between those two groups (*P* = 0.822, 0.229, and 0.298, respectively) (Table 2).

**Table 2:** Expression levels of protein profiles between EAD and non-EAD groups.

	**All (*n* = 105)**	**Non-EAD (*n* = 61)**	**EAD (*n* = 44)**	***P*-value**
High HIF2-α	61 (58.1%)	36 (59.0%)	25 (56.8%)	0.822
High HO1	38 (36.2%)	25 (41.0%)	13 (29.5%)	0.229
High SIRT1	54 (51.4%)	34 (55.7%)	20 (45.5%)	0.298
High TLR4	51 (48.6%)	36 (59.0%)	15 (34.1%)	0.012
High TNF-α	55 (52.4%)	37 (60.7%)	18 (40.9%)	0.046

*EAD, early allograft dysfunction; HIF2-α, hypoxia-inducible factor 2-α; HO1, heme oxygenase-1; SIRT1, sirtuin 1; TLR4, toll like receptor 4; TNF-α, tumor necrosis factor α*.

### Independent Predictors of EAD

To analyze independent predictors of EAD, all five proteins in Table 2 and factors with *P* < 0.1 in Table 1 were included for multivariate logistic regression analysis. The results showed that macrovesicular steatosis ≥ 30% (OR = 0.039, 95% CI = 1.392-25.843, *P* = 0.016), pretransplant serum creatinine (OR = 1.012, 95% CI = 1.001–1.023, *P* = 0.036), and donor BMI (OR = 1.007, 95% CI = 1.002–1.013, *P* = 0.013) were independent predictors of EAD development. Moreover, HO1 (OR = 0.208, 95% CI = 0.059–0.734, *P* = 0.015) and TNF-α (OR = 0.217, 95% CI = 0.051–0.925, *P* = 0.039) were two independent protein markers of the onset of EAD (Table 3). Although lower expression levels of HIF2-α, SIRT1, and TLR4 were observed in the EAD group, they did not reach statistical significance in multivariate analysis (*P* = 0.092, 0.254, and 0.260, respectively).

**Table 3:** Results of multivariate logistic regression analysis of EAD-associated factors.

**Variables**	**OR**	**95% CI**	***P*-value**
HIF2-α	3.190	0.826–12.318	0.092
HO1	0.208	0.059–0.734	0.015
SIRT1	0.509	0.160–1.623	0.254
TLR4	0.520	0.166–1.623	0.260
TNF-α	0.217	0.051–0.925	0.039
Macrovesicular steatosis ≥30%	5.998	1.392–25.843	0.016
CIT	1.157	0.951–1.407	0.145
Serum creatinine	1.012	1.001–1.023	0.036
MELD	1.009	0.958–1.063	0.735
Donor BMI	1.007	1.002–1.013	0.013
Blood loss	1.008	0.986–1.032	0.470

*OR, odds ratio; CI: confident interval; HIF2-α, hypoxia-inducible factor 2-α; HO1, heme oxygenase-1; SIRT1, sirtuin 1; TLR4, toll like receptor 4; TNF-α, tumor necrosis factor α; CIT, cold ischemia time; EAD, early allograft dysfunction; MELD, model for end-stage liver disease score; BMI, body mass index*.

### Survival Analysis

**Figure 2:**
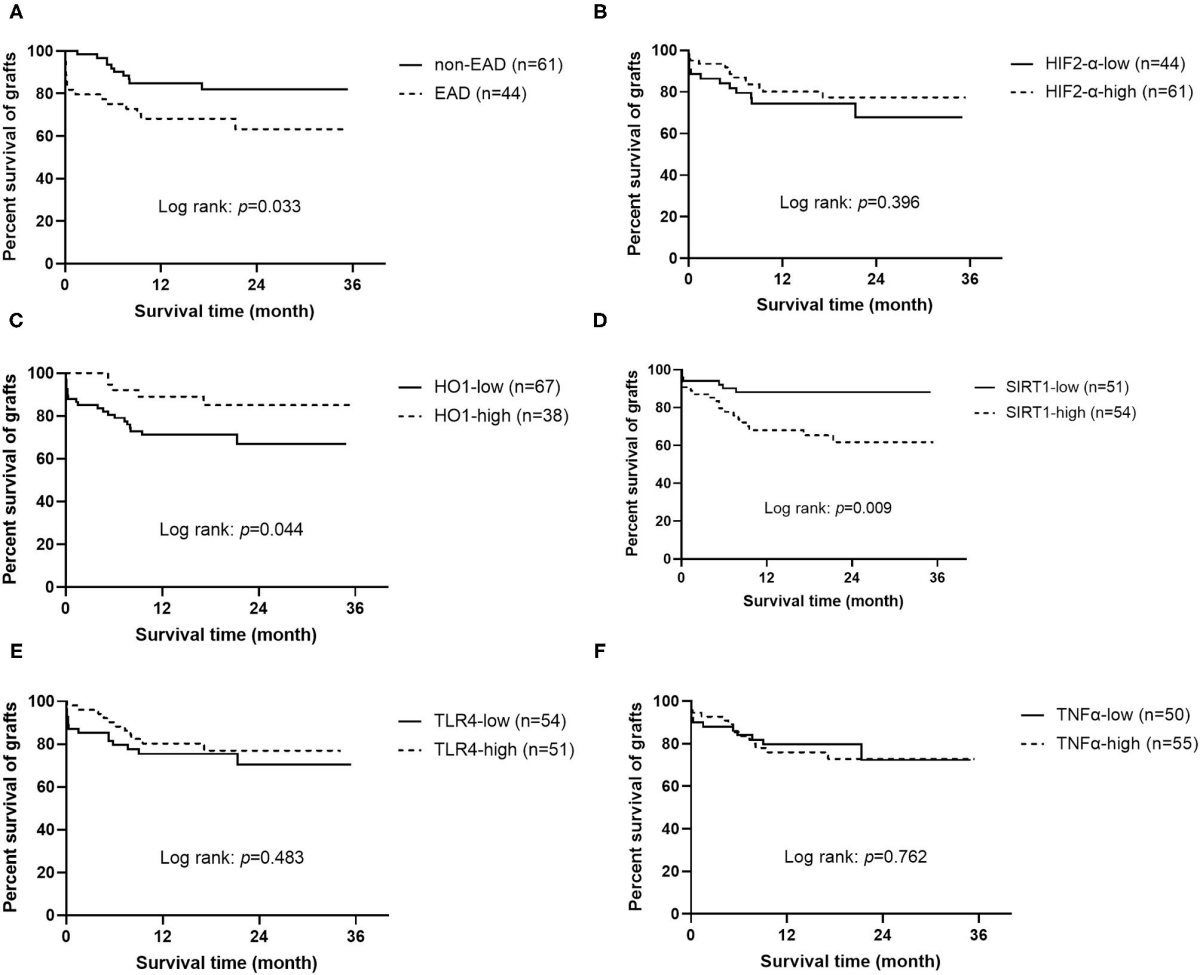
Kaplan-Meier curves for grafts (*n* = 105). **(A)** Graft survival according to EAD or non EAD. **(B)** Graft survival according to the expression level of HIF2-α. **(C)** Graft survival according to the expression level of HO1. **(D)** Graft survival according to the expression level of SIRT1. **(E)** Graft survival according to the expression level of TLR4. **(F)** Graft survival according to the expression level of TNF-α. EAD, early allograft dysfunction; HIF2-α, hypoxia-inducible factor 2-α; HO1, heme oxygenase-1; SIRT1, sirtuin 1; TLR4, toll like receptor 4; TNF-α, tumor necrosis factor α.

To study the effects on graft and recipient survival of EAD and those five protein markers, Kaplan-Meier analysis was performed (Figure 2 and Supplementary Figure 2). Results showed that grafts or recipients who experienced EAD had a worse survival prognosis (log rank = 0.033 and 0.038, respectively). Worse graft survival was also observed in patients who received grafts with lower HO1 expression than in that with higher HO1 expression (log rank = 0.044), but patient survival did not reach statistical significance in those two groups (log rank = 0.089). Interestingly, higher expression level of SIRT1, which showed no correlation with EAD, was identified to be significantly associated with a worse graft and patient survival prognosis (log rank = 0.009 and 0.025, respectively).

Univariate Cox regression analysis confirmed that the expression level of SIRT1 (OR = 3.198, 95% CI = 1.276–8.013, *P* = 0.013), CIT (OR = 1.115, 95% CI = 1.007–1.236, *P* = 0.037), EAD (OR = 2.324, 95% CI = 1.043–5.178, *P* = 0.039), and less blood loss volume (OR = 1.022, 95% CI = 1.011–1.033, *P* < 0.001) was associated with graft survival prognosis. Factors with *P* < 0.1 were further considered for multivariate Cox regression analysis, and results showed that only the expression level of SIRT1 (OR = 6.002, 95% CI = 1.961–18.365, *P* = 0.002) and HO1 (OR = 0.352, 95% CI = 0.125–0.993, *P* = 0.049), EAD (OR = 2.753, 95% CI = 1.117–6.785, *P* = 0.028), and blood loss volume (OR = 1.032, 95% CI = 1.018–1.046, *P* < 0.001) were independent predictors of graft survival (Table 4). In addition, the results of univariate and multivariate Cox regression analysis of patient survival were similar to that of graft survival (Supplementary Table 1), except that HO1 was not an independent predictor of patient survival (OR = 0.381, 95% CI = 0.131–1.114, *P* = 0.078).

**Table 4:** Univariate and multivariate Cox regression analysis of graft survival.

**Variables**	**Univariate analysis**		**Multivariate analysis**	
	**OR (95% CI)**	***P***	**OR (95% CI)**	***P***
HIF2-α	0.714 (0.325–1.565)	0.400	–	–
HO1	0.381 (0.143–1.016)	0.054	0.352 (0.125–0.993)	0.049
SIRT1	3.198 (1.276–8.013)	0.013	4.155 (1.553–11.116)	0.005
TLR4	0.755 (0.343–1.664)	0.486	–	
TNF-α	1.129 (0.512–2.490)	0.763	–	–
CIT	1.115 (1.007–1.236)	0.037	1.034 (0.902–1.184)	0.635
Macrovesicular steatosis ≥30%	1,006 (0.377–2.683)	0.991	–	–
EAD	2.324 (1.043–5.178)	0.039	2.753 (1.117–6.785)	0.028
Serum creatinine	1.003 (1.000–1.006)	0.079	1.002 (0.995–1.008)	0.645
MELD	1.028 (0.997–1.059)	0.076	0.998 (0.955–1.042)	0.913
Donor BMI	0.997 (0.992–1.003)	0.339	–	–
Recipient BMI	0.931 (0.802–1.080)	0.346	–	–
Blood loss	1.022 (1.011–1.033)	<0.001	1.032 (1.018–1.046)	<0.001

*OR, odds ratio; CI: confident interval; HIF2-α, hypoxia-inducible factor 2-α; HO1, heme oxygenase-1; SIRT1, sirtuin 1; TLR4, toll like receptor 4; TNF-α, tumor necrosis factor α; CIT, cold ischemia time; EAD, early allograft dysfunction; MELD, model for end-stage liver disease score; BMI, body mass index*.

### Combination of EAD and Protein Profiles Better Predicts Prognosis of Recipients

**Figure 3:**
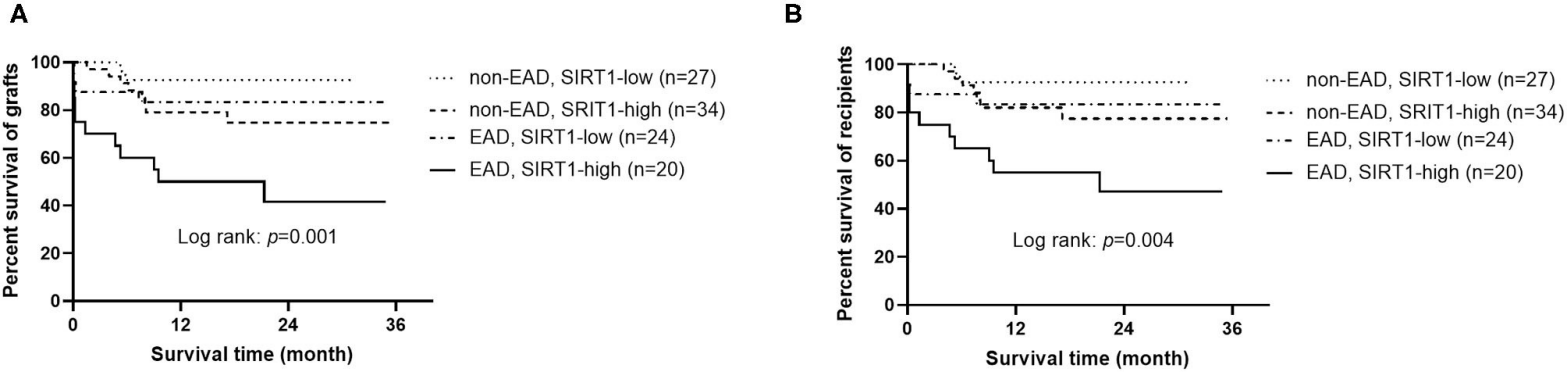
Kaplan-Meier curves for grafts and recipients (*n* = 105). **(A)** Graft survival according to the combination of EAD and SIRT1 expression. **(B)** Recipient survival according to the combination of EAD and SIRT1 expression. EAD, early allograft dysfunction; SIRT1, sirtuin 1.

An interesting phenomenon is that SIRT1 was not associated with the onset of EAD (OR = 0.509, 95% CI = 0.160–1.623, *P* = 0.254), but was a sensitive predictor of graft and patient survival prognosis (OR = 4.155, 95% CI = 1.553–11.116, *P* = 0.005, and OR = 3.559, 95% CI = 1.310–9.669, *P* = 0.013, respectively). To address whether the combination of EAD and SIRT1 was a better predictor for graft or patient survival than each one of them alone, the cases were divided into four groups: (1) patients who received grafts with low expression of SIRT1 and did not develop EAD (*n* = 27); (2) patients who received grafts with high expression of SIRT1 and did not develop EAD (*n* = 34); (3) patients who received grafts with low expression of SIRT1 and developed EAD (*n* = 24); (4) patients who received grafts with high expression of SIRT1 and developed EAD (*n* = 20). As demonstrated in Figure 3, patients with both EAD and higher expression of SIRT1 in grafts had worst graft and patient survival prognosis (*P* = 0.001 and 0.004, respectively).

## Discussion

EAD refers to grafts with initially poor function following liver transplantation, which may deteriorate into primary non-function of grafts, leading to re-transplantation or death. Many factors have been found to contribute to the onset of EAD, including donor age, donor BMI, graft steatosis, CIT, donor serum sodium, and MELD score. Some of these risk factors remain controversial in different studies, which suggests more general, accurate, and reliable predictors of EAD are needed.

Recently, molecular predictors of the onset of EAD have been widely studied. Berberat et al. found that the expression of several mRNAs (CTGF, WWP2, CD274, VEGF, and its receptor FLT1) in post-perfusion grafts was associated with the occurrence of clinical complications following LT in the first month (14). Kurian et al. further identified pathways (PPARα and NF-κB) and targets (i.e., IL-1, CXCL1, TIPARP, TRAF6, and TNFRSF1B) that contribute to the onset of EAD by global gene expression profile analysis (15). Further research has revealed that PPARα is downregulated after LT, which might be an adaptive response to oxidative stress and hepatocellular damage during LT (16). Moreover, microRNAs and circular RNAs, such as miRNA-122, circFOXN2, and circNEXTIN3, that are expressed in the liver have been found to be potential early predictors of the onset of EAD (6, 7). In addition, serum protein profiles of recipients associated with EAD have been widely studied, and several serum proteins have been identified, such as monocyte chemoattractant protein 1, interleukin 8, factor V, and soluble CD163 (8, 9, 17). However, protein profiles in donor grafts associated with EAD are rarely studied. Recently, Xie et al. studied seven proteins expressed in grafts and identified VEGF as an independent risk factor of EAD and survival prognosis (4). In this study, we identified another five proteins in grafts that were associated with EAD, and tried to establish a novel prediction pattern for recipient prognosis, with the combination of EAD and EAD-unrelated proteins, which might be more comprehensive and accurate than using EAD or EAD-related molecules alone.

Hypoxia inducible factors are transcription factors whose expression can be regulated by oxygen supply (10). Since IRI contains an anoxic process, HIF could be upregulated and enter the nucleus, inducing transcription of genes, including VEGF, which has been reported to be associated with EAD development (4). HIF2-α plays a vital role in the development and homeostasis of gastrointestinal tracts, mainly by regulating inflammation, cell metabolism, and fibrosis (10). However, the role of HIF2-α in hepatic graft injury remains unclear. Our results showed that the expression of HIF2-α in liver grafts was not associated with the onset of EAD or survival prognosis of both grafts and recipients.

Heme oxygenase-1 was reported to have anti-oxidative and anti-inflammatory functions (18). A recent study demonstrated that infiltrating mononuclear cells were the major source of HO1 in the liver, while the expression level of HO1 in hepatocytes was low (11), and our results were in accordance with that. A previous study reported that pretransplant high HO1 expression deteriorated graft function after LT (19). On the contrary, according to another study, deteriorated hepatocellular function and survival were observed in LT patients receiving grafts with low macrophage HO1 expression (11). In our results, grafts with low HO1 expression were more likely to develop EAD, which is accordance with the latter study (11). However, an interesting phenomenon was that survival prognosis was worse in grafts with high HO1 expression than in grafts with low HO1 expression, while EAD was less likely to develop in grafts with HO1 expression. This result might be due to complicated confounding factors and/or EAD-unrelated processes that are regulated by HO1, and it implied that EAD or EAD-related molecules alone had limited function in predicting graft and/or recipient prognosis.

Sirtuin 1 is one of the downstream targets of HO1 and plays a vital role in cellular senescence, anti-inflammation, and stress response (20). SIRT1 deletion was reported to aggravate liver IRI (21). Low expression level of SIRT1 was correlated with inferior human hepatic graft function and patient survival (11). SIRT1 was also reported to play a crucial role in hepatocellular senescence, yet few studies have been carried out on discussing the relationship between hepatocellular senescence and the onset of EAD (22–24). Interestingly, in this study, both univariate and multivariate analysis revealed no association between the expression of SIRT1 and the onset of EAD. Considering that HO1 is the upstream regulator of SIRT1 and was associated with EAD, we speculated other pathways including SIRT1 might be involved in the process of EAD development. In addition, we found an interesting phenomenon: high expression of SIRT1 in grafts was significantly correlated with inferior graft and recipient survival. We further studied the combination of EAD and the expression level of SIRT1 in predicting graft and recipient survival. Results showed that grafts or recipients with both EAD and high SIRT1 expression had worst survival prognosis. Therefore, we speculated that combination of EAD and EAD-unrelated molecules might possess more accurate and reliable abilities in predicting graft and recipient survival.

TLR4 was widely reported to have negative effects on liver graft function (25, 26). TLR4-induced liver injury might due to the suppression of downstream HO1 (25), which is a protective factor of hepatic function. Activation of TLR4 could also promote the inflammatory cascade after IRI and played a vital role in gut-liver axis-related liver injury (10). In this study, results showed that TLR4 was not an independent predictor of the onset of EAD, and had no correlation with graft or recipient survival.

TNF-α has critical roles in inflammation and hepatic IRI. Previous animal studies showed that dual-specificity phosphatase 14 could reduce hepatic IRI through quenching TNF-α-induced downstream NF-κB activation (12). TNF-α was also reported to participate in Notch1 or NF-E2-related factor 2-deficiency-induced mouse hepatic IRI (27, 28). In addition, inhibition of hepatic tumor necrosis factor receptor (TRAF) 3 might protect the liver against IRI (29). Previous studies have widely reported that TNF-α, as a proinflammatory molecule, could aggravate hepatic injury. However, in this study, low expression level of TNF-α was observed in grafts experiencing EAD. This phenomenon might be due to the adaptive responses among the process of hepatic IRI (5).

Further optimization of the EAD definition is urgently needed. Previous definitions of EAD established before 2010 are binary, which identified recipients as with or without EAD (3, 25, 30–33). However, those definitions failed to identify patients whose states are near the threshold. Therefore, a continual classification of EAD might better describe the graft situations. The Model for Early Allograft Dysfunction (MEAF) score was thus developed for grading EAD continually, and MEAF score showed advantages in identifying potential EAD patients with parameters mildly below the thresholds of Olthoff's EAD definition (34). Another continuous model named the Liver Graft Assessment Following Transplantation (L-GrAFT) risk score, was established (1). Compared with MEAF score and Olthoff's definition, L-GrAFT risk score showed superiority in predicting 3-month graft failure after LT. Those results showed that there is still room for improvement in the identifying of graft function and predicting of survival prognosis. Until now, little attention has been paid to the expression of proteins in pretransplant grafts, mainly due to uncertain mechanisms and complex processes. Our study identified several EAD-associated proteins, and even EAD-unrelated proteins might also have potential in predicting survival prognosis. Therefore, protein profiles might be crucial for predicting and preventing poor prognosis after LT, and more attention should be paid to this area.

Nevertheless, there were several limitations in this study. First, this is a single-center retrospective study with a relatively small sample size, and we did not have enough cases for developing predicting models and for validation, thus the applicability of these results to other central populations remains to be verified. Second, there were some drawbacks to this technique, for example, the subjective nature of IHC and the fact that a small amount of specimen might be insufficient to evaluate the entire graft. And we recognize that expression patterns of proteins in different zonations of hepatic lobules might be of importance in developing a more accurate model for predicting the onset of EAD and the prognosis after LT. However, that is beyond the scope of this research, and further studies on that question should be carried out in the future. These limitations call for a more accurate and representative technique for evaluating the protein expression patterns of the entire graft, such as quantitative detection of released proteins in preservation solution. Finally, only five potential proteins were analyzed in this study due to the limited amount of specimen. Therefore, further studies should be made to identify the clinical value of other relevant proteins expressed in liver grafts. An integrated protein profile may be useful in guiding the evaluation and management of liver transplantation in the future.

## Conclusions

In conclusion, this study found that pretransplant intrahepatic expression of HO1 and TNF-α are independent predictors of the onset of EAD. And the combination of EAD and intrahepatic SIRT1 might be a superior predictor for graft and patient survival prognosis than using EAD alone.

## Data Availability Statement

The original contributions presented in the study are included in the article/Supplementary Material, further inquiries can be directed to the corresponding author/s.

## Ethics Statement

The studies involving human participants were reviewed and approved by Research Ethics Committee of the First Affiliated Hospital, College of Medicine, Zhejiang University. The patients/participants provided their written informed consent to participate in this study.

## Author Contributions

XX: conception and design. SZ: administrative support. QW, JZ, and KW: data analysis and interpretation. All authors: provision of study material or patients, collection and assembly of data, manuscript writing, and final approval of manuscript.

## Funding

This work was supported by grants from the National Natural Science Foundation of China (Grant Numbers 81800578, 81930016, and 81625003); the Key Research & Development Plan of Zhejiang Province (Grant Numbers 2019C03050 and 2021C03118); and the Projects of Medical and Health Technology Program in Zhejiang Province (Grant Number WKJ-ZJ-2120).

## Conflict of Interest

The authors declare that the research was conducted in the absence of any commercial or financial relationships that could be construed as a potential conflict ofinterest.

## Publisher's Note

All claims expressed in this article are solely those of the authors and do not necessarily represent those of their affiliated organizations, or those of the publisher, the editors and the reviewers. Any product that may be evaluated in this article, or claim that may be made by its manufacturer, is not guaranteed or endorsed by the publisher.
