# Supplementary material for: Combination of Early Allograft Dysfunction and Protein Expression Patterns Predicts Outcome of Liver Transplantation From Donation After Cardiac Death
Source: Front Med (Lausanne). 2021 Dec 8;8:775212. doi: 10.3389/fmed.2021.775212 (PMC8692269; doi:10.3389/fmed.2021.775212)
Supplement: Supplementary file 1 [file Data_Sheet_1.docx]

Supplementary Table 1. Univariate and multivariate Cox regression analysis of patient survival

| Variables | Univariate analysis | | Multivariate analysis | |
| --- | --- | --- | --- | --- |
|  | OR (95% CI) | *P* | OR (95% CI) | *P* |
| HIF2-α | 0.713 (0.314-1.616) | 0.418 | - | - |
| HO1 | 0.434 (0.161-1.170) | 0.099 | 0.381 (0.131-1.114) | 0.078 |
| SIRT1 | 2.767 (1.090-7.023) | 0.032 | 3.559 (1.310-9.669) | 0.013 |
| TLR4 | 0.911 (0.402-2.065) | 0.823 | - | - |
| TNF-α | 1.434 (0.620-3.316) | 0.399 | - | - |
| CIT | 1.071 (0.960-1.195) | 0.219 | - | - |
| Macrovesicular steatosis ≥30% | 1.141 (0.423-3.076) | 0.795 | - | - |
| EAD | 2.364 (1.022-5.469) | 0.044 | 2.760 (1.095-6.956) | 0.031 |
| Serum creatinine | 1.003 (1.000-1.006) | 0.065 | 1.002 (0.997-1.007) | 0.356 |
| MELD | 1.020 (0.988-1.052) | 0.227 | - | - |
| Donor BMI | 0.998 (0.992-1.003) | 0.386 | - | - |
| Recipient BMI | 0.980 (0.846-1.136) | 0.791 | - | - |
| Blood loss | 1.023 (1.012-1.034) | <0.001 | 1.033 (1.018-1.047) | <0.001 |

OR, odds ratio; CI: confident interval; HIF2-α, hypoxia-inducible factor 2-α; HO1, heme oxygenase-1; SIRT1, sirtuin 1; TLR4, toll like receptor 4; TNF-α, tumor necrosis factor α; CIT, cold ischemia time; EAD, early allograft dysfunction; MELD, model for end-stage liver disease score; BMI, body mass index.

**
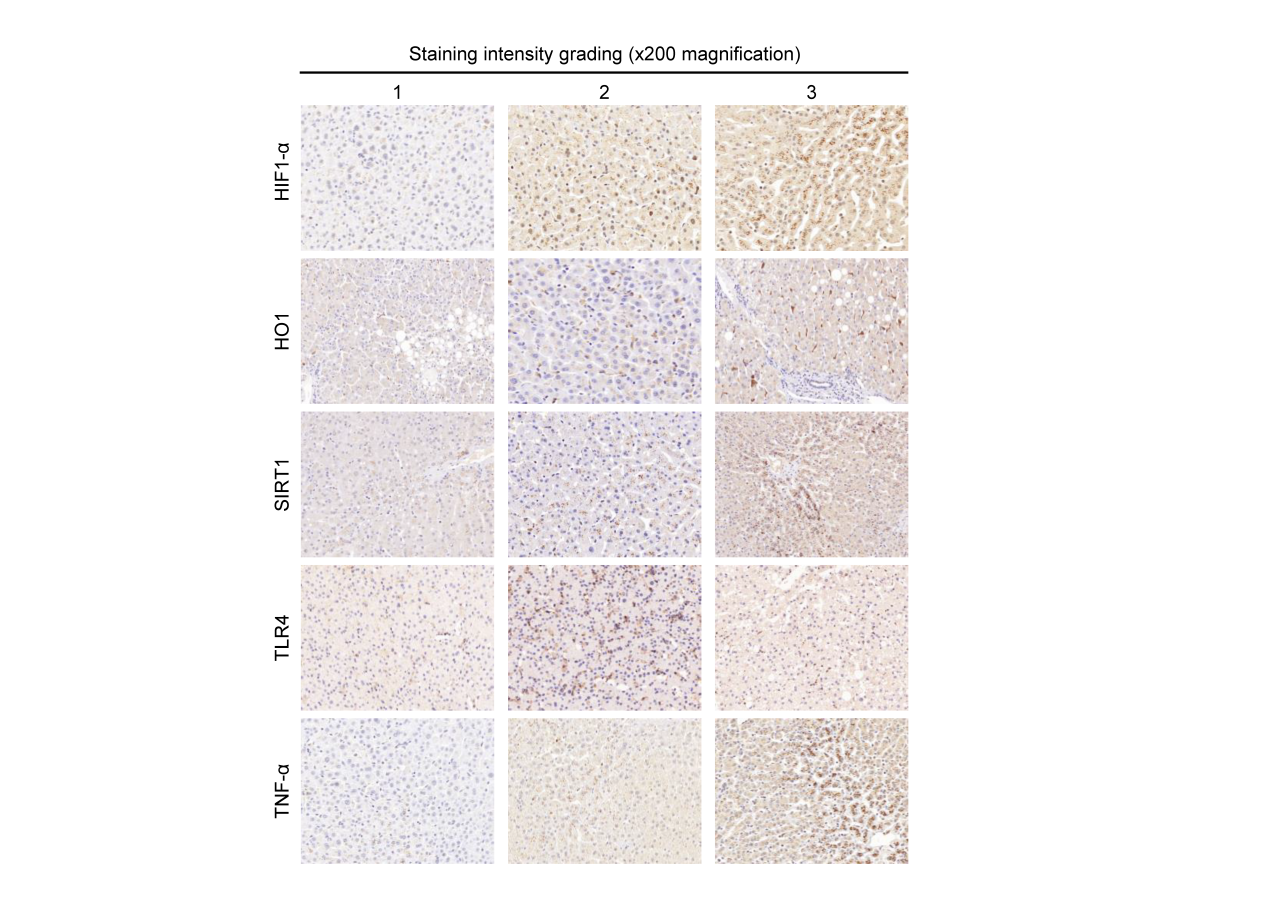
**

Supplementary Figure 1. Representative immunohistochemical results for 3 staining intensity grades (1/2/3) of intrahepatic 5 proteins.


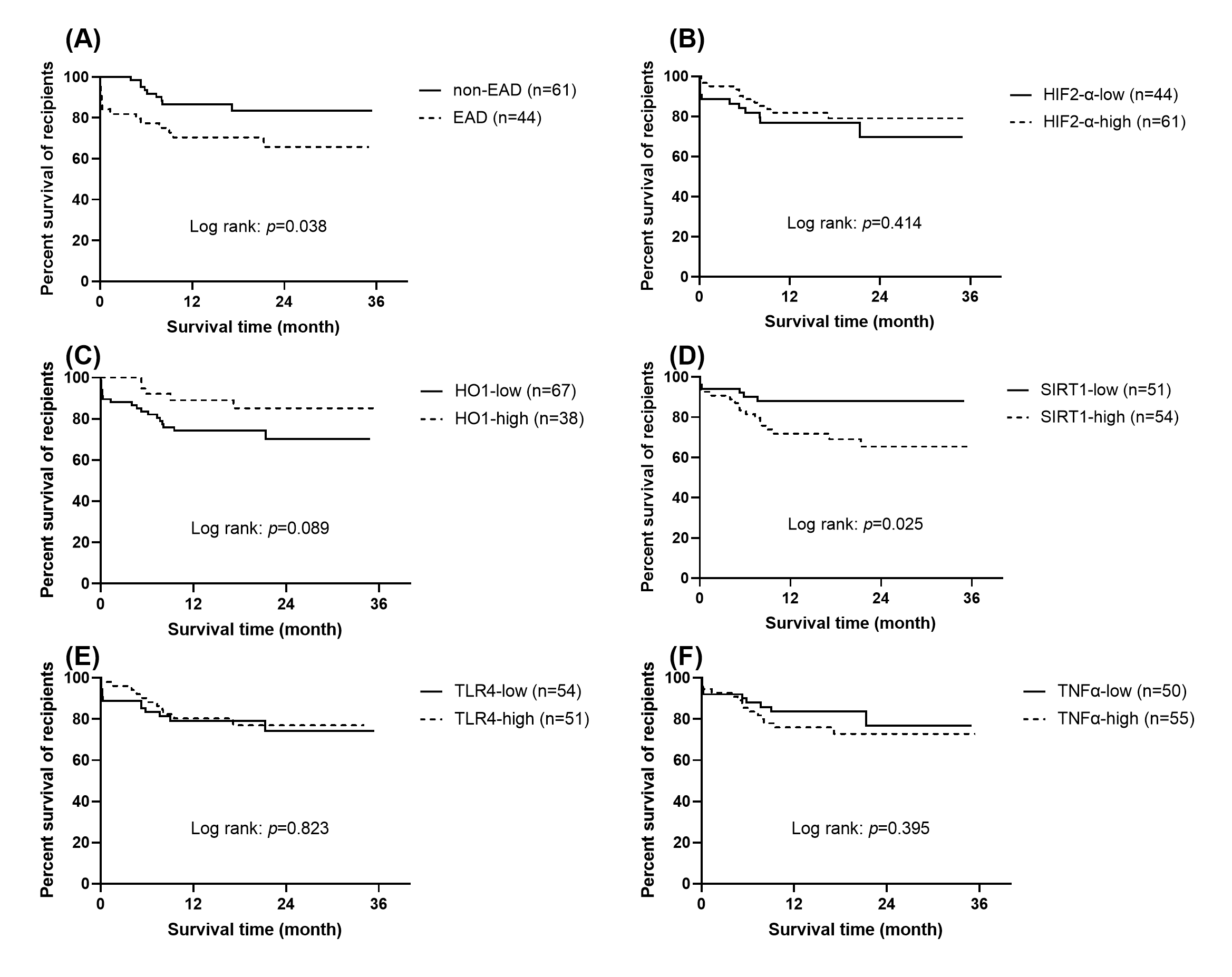


Supplementary Figure 2. Kaplan-Meier curves for recipients. (A) Recipient survival according to EAD or non EAD. (B) Recipient survival according to the expression level of HIF2-α. (C) Recipient survival according to the expression level of HO1. (D) Recipient survival according to the expression level of SIRT1. (E) Recipient survival according to the expression level of TLR4. (F) Recipient survival according to the expression level of TNF-α.

EAD, early allograft dysfunction; HIF2-α, hypoxia-inducible factor 2-α; HO1, heme oxygenase-1; SIRT1, sirtuin 1; TLR4, toll like receptor 4; TNF-α, tumor necrosis factor α.
